# Patient-Facing AI Chatbot Treatment-Direction Advice in Orthodontic Health Communication: A Scenario-Based Comparison with Expert Consensus

**DOI:** 10.3390/healthcare14162565

**Published:** 2026-08-16

**Authors:** Neslihan Karaoğlan, Hakan Karaoğlan

**Affiliations:** 1 Dental Clinic, University of Health Sciences, Sultan II. Abdülhamid Han Training and Research Hospital, 34668 Istanbul, Türkiye; 2 Department of Pediatric Dentistry, University of Health Sciences, 34668 Istanbul, Türkiye; dthakankaraoglan@gmail.com

**Keywords:** artificial intelligence, large language model, chatbot, strategic health communication, AI-mediated health communication, orthodontics, patient education, treatment-direction advice, algorithmic ethics, trust and transparency

## Abstract

**Background/Objectives**: AI chatbots may shape patient expectations before professional consultation. This scenario-based first-response study evaluated whether four user-facing chatbots provided orthodontic treatment-direction advice concordant with an expert benchmark and whether responses contained safety, referral, or overconfidence concerns. **Methods**: Forty fictional Turkish patient-oriented scenarios across eight categories were independently coded by three orthodontists as clear aligners, fixed appliances, both options, examination required, or advanced specialist/surgical evaluation required. Each scenario was submitted once to ChatGPT, Claude, Copilot, and Gemini on 20 May 2026. Two independent non-author orthodontists coded 160 archived first responses using a predefined framework, with adjudication before analysis. **Results**: Inter-expert agreement was moderate (Fleiss kappa = 0.491; Gwet AC1 = 0.528). Under the majority benchmark, exact concordance was 82.5% for ChatGPT, 67.5% for Claude, 42.5% for Copilot, and 37.5% for Gemini (Cochran Q = 34.105, *p* < 0.001). The overall difference remained significant in the 17 unanimous scenarios (Q = 11.455, *p* = 0.010), but a post hoc alternative-reference analysis that adopted the dissenting expert code in the 23 non-unanimous scenarios attenuated the rates to 57.5%, 52.5%, 52.5%, and 42.5%, respectively (Q = 4.222, *p* = 0.238). Coded safety-concern rates ranged from 15.0% to 62.5%. **Conclusions**: The sampled first responses differed in treatment direction and safety coding, but estimates were sensitive to the expert reference definition. Under the tested single-date, single-language, and single-run conditions, the findings represent a conditional snapshot rather than a time-invariant ranking of model capability. Patient-facing chatbots should support nondirective pre-consultation education and referral, not autonomous appliance selection.

## 1. Introduction

Artificial intelligence (AI) is rapidly transforming healthcare communication by changing how health information is produced, personalized, disseminated, and interpreted. Conversational AI systems and large language model-based chatbots are increasingly used by patients to obtain rapid explanations, compare care options, and prepare for clinical encounters. In dentistry and orthodontics, AI is also becoming more visible in digital workflows, patient communication, and educational support [1]. At the same time, patients frequently search for orthodontic information online before consulting a clinician, including information about treatment options such as clear aligners and metal brackets or fixed orthodontic appliances [2,3].

Large language model-based chatbots can provide fluent, accessible, and patient-friendly answers. These characteristics create opportunities for pre-consultation education and may help patients formulate questions for clinicians. However, the same communication features may also create risks when responses are incomplete, overly general, persuasive, or clinically misleading, especially when patients ask questions that require individualized diagnosis and treatment planning [4,5,6,7,8]. In orthodontics, the choice between clear aligners and fixed appliances depends on malocclusion severity, tooth movement complexity, periodontal condition, skeletal pattern, patient compliance, aesthetic expectations, and diagnostic records.

Previous studies and systematic reviews have evaluated the accuracy, reliability, readability, and overall quality of chatbot responses to dental and orthodontic questions [9,10,11,12,13,14,15,16,17,18,19,20,21,22,23,24,25,26,27,28,29,30,31]. Some studies reported an acceptable performance of AI models in answering common orthodontic questions, while others emphasized limitations related to completeness, evidence quality, and clinical safety [3,10,11,12,13,14,15,16,17,18,21,22,23,24,25,26,27,28,29,30,31]. However, most available studies focus on whether chatbot responses are generally correct, informative, or readable. Fewer studies have examined whether AI chatbots may steer patients toward a specific treatment modality, such as clear aligners or fixed appliances, when the information provided by the patient is insufficient for definitive treatment planning.

From a strategic health communication perspective, treatment-direction advice is not merely informational text. It may frame risk, influence the perceived suitability of treatment options, strengthen premature preferences, and affect the patient–clinician discussion that follows. This is ethically relevant because patients may interpret confident AI-generated recommendations as personalized clinical guidance even when the chatbot does not have access to examination findings, radiographs, periodontal assessment, growth evaluation, or full orthodontic records. Safe AI-mediated health communication should therefore support informed decision-making, transparency, and appropriate referral rather than replacing professional assessment.

Contemporary governance frameworks emphasize that health-related AI should preserve human autonomy, promote safety, communicate limitations, and support transparency and accountability. The World Health Organization’s guidance for AI and large multimodal models, the FUTURE-AI consensus framework, and the NIST Generative AI Profile further emphasize explainability, traceability, robustness, pre-deployment evaluation, and lifecycle risk management [32,33,34,35]. For patient-facing chatbots, these principles imply that fluent output should not be treated as sufficient evidence of safe or clinically appropriate decision support.

The Risk Information Seeking and Processing model provides a complementary communication framework: perceived information insufficiency, source trust, and social expectations influence whether individuals continue to seek and systematically process risk information [36]. A confident chatbot response may prematurely reduce perceived information insufficiency and thereby discourage further professional consultation. Similarly, International Patient Decision Aid Standards emphasize the balanced, transparent, and nondirective presentation of options in preference-sensitive decisions [37]. These perspectives support evaluating not only whether an answer is factually plausible, but also whether it frames uncertainty and treatment options without premature steering.

Therefore, this study aimed to compare the treatment-direction recommendations of four AI chatbots in response to standardized Turkish patient-oriented orthodontic scenarios. AI recommendations were compared with a clinically reasoned expert consensus benchmark established by three orthodontic specialists. The primary outcome was exact concordance between the AI-generated treatment recommendation and the expert consensus decision. Secondary analyses interpreted exact concordance together with safety coding, referral behavior, overconfident wording, and clear-aligner over-direction. The null hypothesis was that there would be no significant difference among the evaluated AI chatbots in their concordance with expert consensus.

## 2. Materials and Methods

### 2.1. Study Design and Ethical Considerations

This study was designed as a cross-sectional, scenario-based comparative evaluation of AI chatbot responses to patient-oriented orthodontic treatment-selection questions. The study assessed whether AI chatbots directed patients toward clear aligner therapy, metal bracket/fixed appliance treatment, both treatment options, clinical examination before decision-making, or advanced specialist/surgical evaluation. The scenarios were treated as simulated AI-mediated health communication encounters in which a patient seeks treatment-direction advice before professional orthodontic consultation.

The study did not recruit or interact with patients or other individuals as research participants and did not use patient records, identifiable personal data, clinical interventions, animals, or biological materials. All scenarios were fictional, and the primary objects of evaluation were archived AI-generated text outputs. External orthodontic experts and independent coders provided non-identifiable professional assessments of fictional scenarios and anonymized AI responses; no personal, health-related, or identifiable information was collected from them. On the basis of these study characteristics and the applicable institutional requirements, the authors determined that the project fell outside the scope of formal human-subject ethics review. Because there was no participant recruitment, intervention, or collection of identifiable data, no application for formal approval or an exemption letter was submitted. This statement reports the basis of the authors’ determination and does not represent a committee-issued exemption.

### 2.2. Development of Clinical Scenarios

A total of 40 fictional orthodontic clinical scenarios were developed to represent common and clinically relevant situations in which patients may compare clear aligner therapy and metal bracket/fixed appliance treatment. The scenarios were grouped into eight categories, with five scenarios in each category: mild orthodontic problems, moderate or borderline cases, severe crowding and rotations, vertical problems, sagittal problems, patient compliance and lifestyle-related factors, risky or complex clinical conditions, and aesthetic expectations or patient preference.

Scenario development followed an investigator-led four-stage process. First, an a priori content blueprint was created to cover eight clinically relevant treatment-selection domains. Second, candidate vignettes were drafted to vary crowding severity, rotation, vertical and sagittal relationships, compliance, periodontal or other biological risk, aesthetic preference, and possible need for advanced evaluation. Third, the investigators reviewed the candidate pool for duplication, category coverage, internal consistency, and patient-facing clarity. Fourth, the final five scenarios per category were selected to provide a balanced spectrum of decision difficulty rather than to estimate population prevalence. The scenarios underwent iterative investigator review, but no formal content-validity index or independent lay-user pilot test was performed; this is acknowledged as a limitation.

The number of scenarios was determined pragmatically to balance clinical breadth and feasibility. Eight prespecified categories with five scenarios each allowed within-scenario comparison across four models; category-level analyses were designated exploratory. No a priori power calculation was performed because no established clinically meaningful paired difference was available for this treatment-direction outcome and the corpus was constructed around domain coverage. To contextualize precision, an approximate paired-binary minimum-detectable-effect calculation for *n* = 40, 80% power, and two-sided alpha = 0.05 indicated detectable absolute paired differences of approximately 22 percentage points when 25% of pairs were discordant and 31 percentage points when 50% were discordant; multiplicity adjustment would require larger differences. Accordingly, non-significant pairwise findings were not interpreted as evidence of equivalence.

For each scenario, two versions were prepared: a standardized clinical vignette for the expert panel and a natural-language Turkish patient-facing prompt for the chatbots. This preserved a common clinical core while approximating how patients may ask treatment-related questions. One standardized wording was used per scenario to ensure identical model inputs; no paraphrased-prompt sensitivity analysis was conducted. The complete vignettes and prompts are provided in Supplementary File S1.

### 2.3. Expert Consensus Reference Standard

Three orthodontic specialists, each with at least 8 years of clinical experience, independently evaluated the 40 standardized clinical scenarios before reviewing any AI-generated responses. For each scenario, the experts selected one of the following five treatment-direction categories: (1) clear aligner therapy may be more appropriate; (2) metal bracket/fixed appliance treatment may be more appropriate; (3) both treatment options may be appropriate; (4) no decision can be made without clinical examination; and (5) advanced specialist and/or surgical evaluation is required. The category “both treatment options may be appropriate” was used when the written scenario contained sufficient information to consider both clear aligners and fixed appliances as clinically plausible options. In contrast, “no decision can be made without clinical examination” was used when additional clinical, radiographic, periodontal, skeletal, or functional assessment would be required before any appliance-direction recommendation. Expert consensus codes are provided in Supplementary File S2.

The operational reference code for each scenario was determined by majority agreement. If all three experts had selected different categories, a consensus discussion would have been planned; no scenario required this step because a 2/3 or unanimous decision was available in all 40 scenarios. No attempt was made to force unanimity after independent coding. Individual expert codes, including every dissenting code, were retained in Supplementary File S2, and unanimous and 2/3-majority scenarios were reported separately. Because the benchmark was based on written vignettes rather than full records, it was treated as a clinically reasoned reference rather than an absolute diagnostic gold standard. Additional analyses compared the models with the unanimous subset, with all individual expert judgments descriptively, and with an alternative reference that substituted the dissenting expert code in the 23 non-unanimous scenarios.

### 2.4. AI Chatbot Response Generation

Four chatbots were evaluated through the publicly available user-facing interfaces accessible to the authors at data collection: ChatGPT, GPT-5.5 Thinking; Claude, Claude Sonnet 5; Gemini, Gemini 3.5 Flash; and Microsoft Copilot (Smart mode, web version; Microsoft Corporation, Redmond, WA, USA), Smart mode. Displayed model or interface-mode names were recorded exactly as shown to the user. Microsoft Copilot did not disclose the underlying foundation-model identifier for Smart mode; therefore, the Copilot findings apply to that user-facing mode on the stated date and cannot be attributed to a named underlying model. This lack of backend specification limits exact replication if the interface or its model assignment changes.

All patient-facing prompts were entered in Turkish on 20 May 2026 between 14:00 and 18:00 local time in Türkiye. Browsing/search, memory, custom instructions, role assignment, additional context, follow-up prompts, and response regeneration were not used. Each prompt was submitted in a separate new chat, and only the first response was archived. This first-response design was intended to represent the initial answer a patient might encounter, but it does not estimate within-model stochastic variability. Repeated runs and paraphrased prompts were not performed; therefore, observed outputs may vary under repeated generation or alternative wording. Verbatim Turkish responses are provided in Supplementary File S3.

### 2.5. Coding of AI-Generated Responses

Each AI response was coded according to a predefined categorical treatment-direction framework. The AI recommendation code was assigned as follows: (1) clear aligner therapy may be more appropriate; (2) metal bracket/fixed appliance treatment may be more appropriate; (3) both treatment options may be appropriate; (4) no decision can be made without clinical examination; (5) advanced specialist and/or surgical evaluation is required; and (6) unclear or not classifiable. The coded dataset is provided as Supplementary File S4, and the coding framework is provided as Supplementary File S5. AI-generated responses were coded by two independent orthodontists who were not authors of the manuscript and were not involved in scenario development, expert benchmark construction, or AI response generation. Before formal coding, both coders received the same written coding framework and operational definitions; no expert consensus codes were disclosed during this stage. During the initial coding process, the coders were blinded to the expert consensus reference codes and coded the AI-generated responses independently using the predefined framework. Treatment-direction coding followed a dominant-direction hierarchy: if a response included an examination caveat but still conveyed a dominant appliance preference, it was coded according to that dominant treatment direction, and the examination caveat was captured separately as a referral-to-examination variable. Code 4 was assigned only when the response avoided a dominant appliance preference and stated that no treatment-direction decision could be made safely before clinical examination and diagnostic records. Code 5 was assigned when the response identified advanced specialist assessment and/or possible surgical evaluation as necessary before appliance selection. If a response gave a definitive treatment direction despite insufficient clinical information, it was coded according to the stated treatment direction and was additionally evaluated for overconfident wording and safety concern. Disagreements between the two coders were reviewed and resolved by consensus/adjudication before final statistical analysis. To reduce bias, chatbot identifiers were removed from coding files where feasible; however, complete blinding to model identity could not be guaranteed because stylistic and content features may reveal the source model.

The primary outcome was exact concordance between the AI recommendation code and the expert consensus benchmark. Responses were classified as concordant if the AI recommendation code exactly matched the expert consensus code and discordant if it did not. Exact concordance was selected as the primary outcome because the study focused on treatment-direction alignment; however, discordant responses were interpreted together with safety coding because some discordant responses, such as recommending clinical examination when both options could be possible, may be clinically cautious rather than harmful.

Clinical safety was evaluated using a three-level framework: 0 = clinically appropriate and balanced; 1 = incomplete but not directly unsafe; and 2 = potentially misleading or clinically inappropriate treatment-direction advice. A coded safety concern was defined as an adjudicated final safety code of 2. Referral to orthodontic examination and definite or overconfident wording were recorded as descriptive binary features. Clear-aligner over-direction was defined as an AI clear-aligner recommendation when the majority expert benchmark did not favor clear aligners; non-safety-concern discordance was defined as discordance with safety code 0 or 1.

To improve clinical granularity without changing the original 0/1/2 safety scale, post hoc, non-mutually exclusive descriptive mechanism indicators were derived after adjudication from the final recommendation and safety codes, expert category, scenario domain, and existing coder flags. These indicators were not prespecified before data collection and were treated as exploratory descriptive summaries rather than confirmatory coding outcomes. They included clear-aligner over-direction among safety-concern responses; under-referral when the expert benchmark required examination or advanced evaluation (codes 4 or 5) but the AI recommendation did not; missed advanced referral when expert code 5 was not identified; overconfident wording when either coder flagged this feature; safety concerns in biomechanical or skeletal scenarios (scenario IDs 11–25); and safety concerns in risky or complex clinical scenarios (scenario IDs 31–35). The indicators could overlap.

### 2.6. Statistical Analysis

Statistical analyses were performed using Python 3.11 with pandas 2.2.3, NumPy 2.3.5, SciPy 1.17.0, statsmodels 0.14.6, and scikit-learn 1.8.0. Categorical variables were summarized as frequencies and percentages. Wilson 95% confidence intervals were calculated for model-specific concordance and coded safety-concern rates. Cohen’s kappa values comparing each model with the majority expert benchmark were accompanied by scenario-level nonparametric bootstrap 95% confidence intervals. These intervals quantify variation across the scenario set and do not include unmeasured run-to-run generation variability. Inter-coder reliability was assessed using observed agreement and Cohen’s kappa for treatment-direction and safety coding; quadratic weighted kappa was also calculated for the ordinal safety scale.

Inter-expert agreement across the five nominal treatment-direction categories was assessed using Fleiss’ kappa and Gwet AC1. Pairwise agreement between experts was additionally assessed using Cohen’s kappa. Because the expert benchmark was based on written scenarios rather than complete clinical records, the level of expert agreement was reported to contextualize subsequent model-concordance results. Results were also stratified by expert agreement level (unanimous vs. 2/3 majority scenarios).

The primary analysis compared exact concordance across the four chatbots using Cochran’s Q test because the same 40 scenarios were evaluated by each model. Significant omnibus tests were followed by pairwise exact McNemar tests with Holm adjustment within each outcome family. The same procedure compared coded safety-concern rates. For each pairwise comparison, discordant-pair counts, paired risk differences in percentage points, and matched-pairs odds ratios with exact 95% confidence intervals were reported. The matched odds ratio was calculated from the two discordant cells; when the denominator cell was zero, the estimate was reported as infinity with its exact lower confidence bound.

Reference-standard sensitivity was evaluated in three ways: stratification by unanimous versus 2/3-majority expert agreement; descriptive concordance with each individual expert and aggregate support across the 120 expert-scenario judgments; and an additional post hoc alternative-reference analysis in which the dissenting expert code replaced the majority code in each of the 23 non-unanimous scenarios while unanimous codes were retained. Cochran’s Q and Holm-adjusted exact McNemar tests were repeated for the alternative binary concordance outcome. These analyses were used to assess dependence of the model comparison on the operational reference definition.

**Figure 1:**
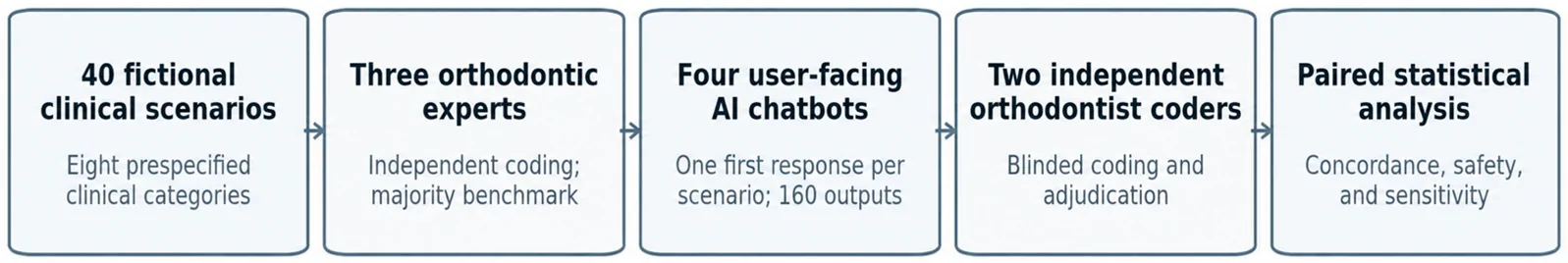
Study workflow from scenario development to paired statistical analysis.

Model-specific recommendation patterns were summarized descriptively and compared with the expert distribution. Category-based analyses were exploratory because each category included five scenarios. Additional analyses separated exact concordance, discordance without coded safety concern, potentially unsafe discordance, clear-aligner over-direction, and the non-mutually-exclusive safety mechanisms defined above. A two-sided *p* value < 0.05 was considered statistically significant. The study workflow is summarized in Figure 1.

## 3. Results

### 3.1. Expert Consensus and Inter-Expert Agreement

A final expert consensus treatment-direction code was obtained for all 40 scenarios. The most frequent consensus category was no decision can be made without clinical examination, in 17 scenarios (42.5%), followed by metal bracket/fixed appliance treatment may be more appropriate in 8 scenarios (20.0%), clear aligner therapy may be more appropriate in 7 scenarios (17.5%), both treatment options may be appropriate in 6 scenarios (15.0%), and advanced specialist and/or surgical evaluation is required in 2 scenarios (5.0%).

Among the 40 scenarios, complete agreement among all three experts was observed in 17 scenarios, while a 2/3 majority agreement was observed in 23 scenarios. No scenario had three different expert decisions. Overall inter-expert agreement was moderate, with a Fleiss kappa value of 0.491 and a Gwet AC1 value of 0.528. The mean observed agreement was 0.617, and pairwise Cohen kappa values ranged from 0.283 to 0.757, indicating that several treatment-selection scenarios were clinically borderline even for specialists. Therefore, AI concordance results should be interpreted as alignment with a clinically reasoned consensus benchmark rather than as agreement with an absolute diagnostic truth. Scenarios with 2/3 majority agreement are identified in Supplementary File S2 to support transparency regarding clinically ambiguous cases. The expert consensus distribution and inter-expert agreement statistics are summarized in Table 1.

Inter-coder reliability for AI response coding was substantial. For treatment-direction coding, the two independent orthodontist coders showed exact agreement in 144/160 responses (90.0%), with a Cohen’s kappa of 0.857. For the three-level safety coding, exact agreement was observed in 135/160 responses (84.4%), with a Cohen’s kappa of 0.756 and a quadratic weighted kappa of 0.846. Overall, 30/160 responses (18.8%) required review/adjudication before final analysis. Model-specific review/adjudication was required for 5/40 ChatGPT responses (12.5%), 6/40 Claude responses (15.0%), 3/40 Copilot responses (7.5%), and 16/40 Gemini responses (40.0%).

### 3.2. Overall AI Performance

Across 160 archived first responses, 92 (57.5%; 95% CI: 49.8–64.9%) exactly matched the majority expert benchmark and 56 (35.0%; 95% CI: 28.0–42.7%) received an adjudicated safety code of 2. Of 68 discordant responses, 49 (72.1%) were coded as safety concerns and 19 (27.9%) were discordant without a coded safety concern. Referral and overconfidence remained descriptive response features.

### 3.3. Model-Specific Concordance with Expert Consensus

**Figure 2:**
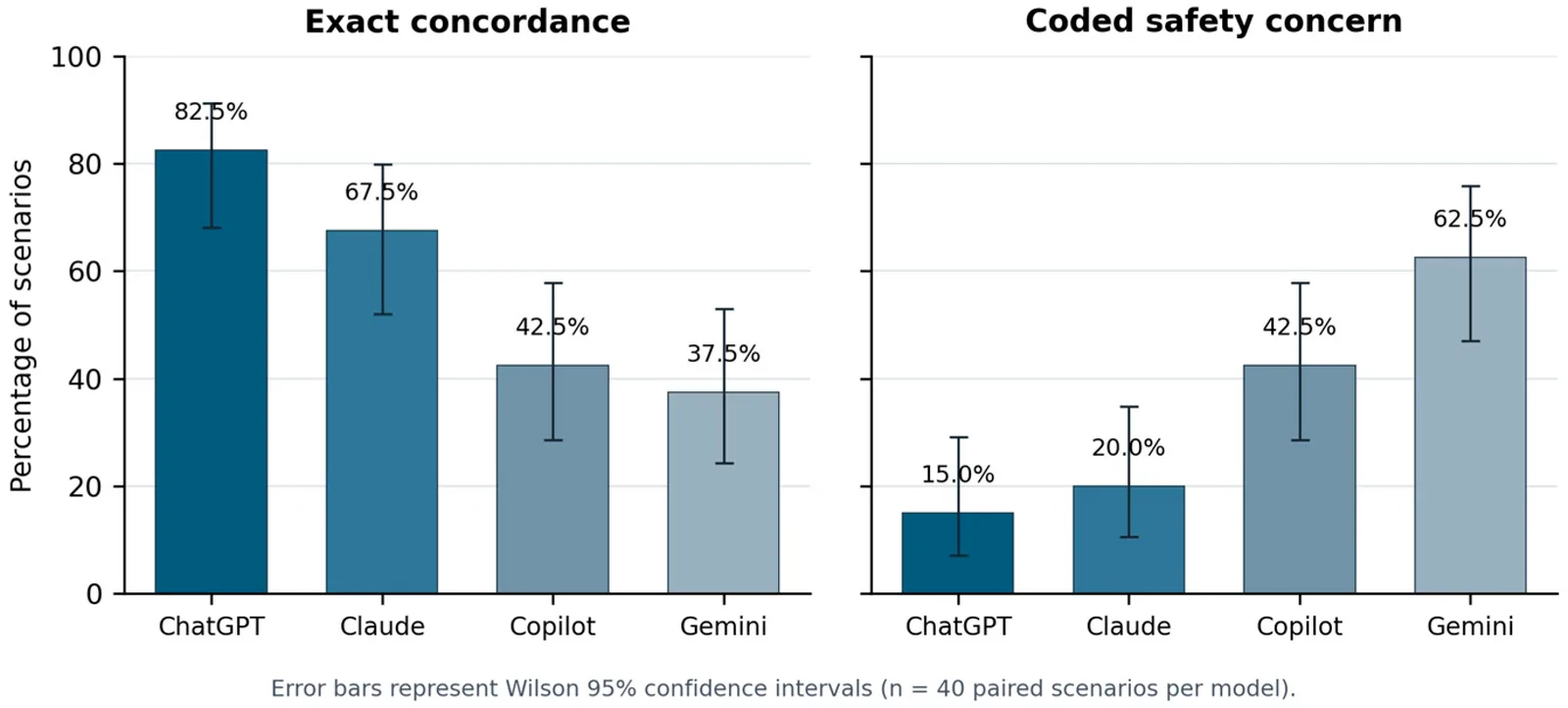
Model-specific exact concordance and coded safety-concern rates with Wilson 95% confidence intervals.

Under the majority expert benchmark, observed exact concordance was 33/40 (82.5%; 95% CI: 68.1–91.3%) for ChatGPT, 27/40 (67.5%; 52.0–79.9%) for Claude, 17/40 (42.5%; 28.5–57.8%) for Copilot, and 15/40 (37.5%; 24.2–53.0%) for Gemini (Table 2 and Figure 2). These values describe the sampled first responses under the stated conditions.

Cohen’s kappa against the majority benchmark was 0.757 (bootstrap 95% CI: 0.586–0.897) for ChatGPT, 0.562 (0.361–0.748) for Claude, 0.294 (0.148–0.444) for Copilot, and 0.225 (0.087–0.388) for Gemini. These estimates should be interpreted with the category distribution and moderate inter-expert agreement rather than as agreement with an absolute clinical truth.

Exact concordance differed across models under the majority benchmark (Cochran Q = 34.105, df = 3, *p* < 0.001). Holm-adjusted McNemar tests showed higher observed concordance for ChatGPT than Copilot and Gemini and for Claude than Copilot and Gemini; ChatGPT versus Claude and Copilot versus Gemini were not significant. Discordant-pair counts, paired risk differences, matched-pairs odds ratios with exact 95% confidence intervals, and adjusted *p* values are reported in Table 3.

Agreement-stratified analyses are summarized in Table 4. In unanimous scenarios (*n* = 17), exact concordance was 17/17 (100.0%) for ChatGPT, 14/17 (82.4%) for Claude, 12/17 (70.6%) for Copilot, and 11/17 (64.7%) for Gemini (Cochran Q = 11.455, *p* = 0.010). In 2/3-majority scenarios *(n* = 23), the corresponding rates were 16/23 (69.6%), 13/23 (56.5%), 5/23 (21.7%), and 4/23 (17.4%) (Q = 23.333, *p* < 0.001). However, when the dissenting expert code replaced the majority code in all 23 non-unanimous scenarios, concordance attenuated to 23/40 (57.5%), 21/40 (52.5%), 21/40 (52.5%), and 17/40 (42.5%), respectively, and the omnibus difference was not significant (Q = 4.222, *p* = 0.238). Descriptively, across all 120 individual expert-scenario judgments, the models matched 89 (74.2%), 75 (62.5%), 55 (45.8%), and 47 (39.2%) judgments, respectively. Thus, aggregate expert support resembled the majority-benchmark ordering, but formal model separation was sensitive to the reference definition.

### 3.4. Treatment-Direction Recommendation Patterns

The distribution of AI treatment-direction recommendations differed across models and was compared descriptively with the expert consensus distribution. ChatGPT most closely reproduced the expert pattern for examination-required cases, selecting no decision can be made without clinical examination in 17 responses (42.5%). Claude selected clear aligner therapy in 14 responses (35.0%) and examination required in 14 responses (35.0%). Copilot selected clear aligner therapy in 19 responses (47.5%) and metal bracket/fixed appliance treatment in 15 responses (37.5%), while selecting examination required in only 1 response (2.5%). Gemini showed the strongest tendency toward clear aligner recommendation, selecting clear aligner therapy in 29 responses (72.5%).

Compared with the expert consensus distribution, the most clinically relevant descriptive patterns were the low rate of examination-required recommendations by Copilot and the high rate of clear-aligner direction by Gemini. Clear-aligner over-direction, defined descriptively as an AI clear-aligner recommendation when the expert consensus was not clear aligner therapy, occurred in 4/40 ChatGPT responses, 7/40 Claude responses, 12/40 Copilot responses, and 22/40 Gemini responses. Among the 19 scenarios in which the expert consensus required clinical examination or advanced/surgical evaluation, the AI recommendation code failed to indicate either examination or advanced evaluation in 2/19 ChatGPT responses, 6/19 Claude responses, 17/19 Copilot responses, and 16/19 Gemini responses. These patterns help explain why exact concordance and coded safety-concern rates differed among models.

Model-specific confusion matrices comparing expert consensus categories with adjudicated AI recommendation codes are provided in Supplementary File S4. These matrices further identify whether discordance occurred as premature clear-aligner direction, fixed-appliance direction, under-referral for clinical examination, or failure to identify advanced specialist/surgical evaluation needs. The distributions of expert consensus and model-specific treatment-direction recommendations are presented in Table 5.

### 3.5. Interpretive Analysis Combining Exact Concordance and Safety Coding

Because exact concordance may underestimate clinically cautious responses, an additional descriptive analysis separated discordant responses without a coded safety concern from potentially unsafe discordance. Responses without coded safety concern were most frequent for ChatGPT (34/40, 85.0%) and Claude (32/40, 80.0%), followed by Copilot (23/40, 57.5%) and Gemini (15/40, 37.5%). Potentially unsafe discordance followed the opposite pattern, with 2/40 ChatGPT responses, 7/40 Claude responses, 17/40 Copilot responses, and 23/40 Gemini responses coded as discordant safety concerns. This analysis indicates that some non-matching responses were clinically cautious rather than unsafe, whereas model differences remained pronounced when discordance was interpreted together with safety coding. The combined analysis of exact concordance, non-safety-concern discordance, potentially unsafe discordance, and clear-aligner over-direction is summarized in Table 6.

### 3.6. Safety Concerns

Adjudicated safety-code-2 rates were 6/40 (15.0%; 95% CI: 7.1–29.1%) for ChatGPT, 8/40 (20.0%; 10.5–34.8%) for Claude, 17/40 (42.5%; 28.5–57.8%) for Copilot, and 25/40 (62.5%; 47.0–75.8%) for Gemini (Table 2 and Figure 2).

Safety-concern rates differed across models (Cochran Q = 32.093, df = 3, *p* < 0.001). Holm-adjusted McNemar results and matched-pairs odds ratios are reported in Table 3. Descriptive mechanism coding showed that under-referral in examination/advanced-evaluation scenarios accounted for 2, 5, 16, and 15 safety-concern responses for ChatGPT, Claude, Copilot, and Gemini, respectively; clear-aligner over-direction among safety concerns occurred in 1, 2, 7, and 21 responses. Overconfident wording was identified by at least one coder in 2, 3, 0, and 19 safety-concern responses. Mechanisms were non-mutually exclusive and are summarized in Table 7; representative translated examples remain in Supplementary File S6.

### 3.7. Exploratory Category-Based Findings

Category-based analyses were interpreted as exploratory because each category included only five scenarios. All four models achieved complete concordance in mild orthodontic problems (5/5). Differences between models became more apparent in clinically more complex categories. ChatGPT showed consistently high concordance in moderate/borderline, risky/complex, and aesthetic-preference scenarios. Gemini showed particularly low concordance in vertical and sagittal problem scenarios. Exploratory category-specific concordance results are presented in Table 8.

## 4. Discussion

### 4.1. Principal Findings and Comparison with Previous Studies

This study compared the archived first-response treatment directions of four patient-facing chatbots under standardized Turkish-language conditions. Exact concordance and safety coding differed under the majority expert benchmark, but the magnitude and statistical separation depended on how non-unanimous expert judgments were operationalized. The overall difference persisted in the unanimous subset but was attenuated and non-significant when the dissenting expert codes were used as the alternative reference. The findings therefore characterize conditional output patterns under the sampled interface, prompt, language, date, and first-run conditions; they do not establish a time-invariant ranking of underlying model capability.

Viewed through a strategic health communication lens, chatbot responses function as persuasive patient-facing messages rather than neutral technical outputs. In RISP terms, a fluent and confident answer may prematurely reduce perceived information insufficiency and discourage further information seeking [36]. From a shared-decision and IPDAS perspective, pre-consultation information should present options, limitations, benefits, harms, and uncertainty in a balanced manner without steering a preference-sensitive choice before clinical assessment [37]. Safety therefore depends not only on factual plausibility, but also on whether uncertainty and the need for examination are communicated clearly.

The results are consistent with previous studies showing that AI performance in orthodontics is model-dependent. Daraqel et al. compared ChatGPT and Google Bard for general orthodontic questions and reported differences in response performance between models [3]. Similar model-dependent variability has also been reported in studies evaluating ChatGPT, Bard, Bing, Gemini, Copilot, and other chatbot systems in orthodontic contexts [19,20,24,25,27,28]. These findings are also consistent with recent systematic evidence showing that the orthodontic performance of LLMs varies across models, question types, and evaluation methods [9].

The higher observed majority-benchmark concordance for ChatGPT and Claude is consistent with reports that newer LLM interfaces can provide generally acceptable answers to common orthodontic questions [12,13,14,15,16,17,18,26,31]. Nevertheless, the reference-sensitivity analysis shows why these values should not be treated as stable capability rankings. A fluent answer can remain clinically problematic if it directs a patient toward an appliance that expert judgments do not consistently support or that cannot be selected without examination. Patient-facing systems may therefore be more appropriate as pre-consultation education tools that promote assessment and shared decision-making than as autonomous treatment-selection advisors.

### 4.2. Clinical, Health Communication, and Governance Implications

A notable finding was the tendency of some models, particularly Gemini, to recommend clear aligner therapy more frequently than the expert consensus supported. This has potential communication and clinical implications because clear aligner therapy is highly dependent on case selection, patient compliance, attachment planning, staging, tracking, refinements, and the predictability of specific tooth movements. Previous studies evaluating AI responses about clear aligners have shown that responses may be incomplete or insufficiently nuanced, especially when questions involve indications, limitations, or complex biomechanics [10,11,19]. In the present study, excessive or premature clear-aligner direction was particularly problematic in scenarios involving severe crowding, vertical problems, sagittal discrepancies, compliance uncertainty, periodontal risk, or possible surgical need. Such over-direction may reinforce patient expectations before professional consultation and may complicate shared decision-making if patients arrive with a premature preference shaped by AI-generated advice.

The coded safety findings further reinforce the need for caution. Responses coded as potentially misleading or clinically inappropriate generally reflected overly definitive treatment-direction advice, insufficient emphasis on clinical examination, or inappropriate reassurance in complex scenarios. In health communication terms, such responses may operate as a form of overconfident risk communication: they reduce uncertainty where uncertainty should be preserved and may make a complex clinical decision appear simpler than it is. The distinction between exact concordance and non-safety-concern discordance is therefore clinically important. Some discordant answers were not necessarily unsafe, particularly when they recommended examination rather than a specific appliance. Conversely, a definitive clear-aligner or fixed-appliance recommendation in a diagnostically insufficient or complex scenario may be more concerning than a cautious non-matching response.

The rate of referral to orthodontic examination also differed across models. ChatGPT most frequently emphasized the need for clinical examination or orthodontist consultation, whereas Copilot did so least often. This difference is meaningful because many orthodontic treatment decisions cannot be made from a short text-based patient description. Radiographs, intraoral examination, digital models, periodontal assessment, growth evaluation, and facial analysis may all be required. This is particularly relevant in scenarios involving impacted canines, skeletal Class III tendency, deep bite requiring intrusion mechanics, open bite with possible tongue thrust, periodontal compromise, root resorption history, or implant-related planning. Safe AI-assisted patient education should prioritize triage, risk recognition, transparency about diagnostic limitations, and referral to orthodontic examination over appliance selection.

The findings also raise governance considerations for AI-mediated health communication. WHO guidance emphasizes protection of autonomy, well-being, safety, transparency, intelligibility, responsibility, and accountability [32,33]. FUTURE-AI and the NIST Generative AI Profile additionally emphasize traceability, robustness, explainability, pre-deployment testing, monitoring, and lifecycle risk management [34,35]. In this context, patient-facing chatbots should communicate diagnostic limitations, avoid converting uncertainty into definitive treatment advice, and direct users to qualified clinicians when examination or advanced evaluation is required. Evaluation beyond English is also necessary because linguistic nuance and local care expectations may influence interpretation and trust.

The present findings help clarify the novelty of this study in relation to the existing literature. Many previous orthodontic AI studies have assessed accuracy, completeness, readability, evidence quality, reliability, or comparison with clinician responses [3,9,10,11,12,13,14,15,16,17,18,19,20,21,22,23,24,25,26,27,28,29,30,31]. Fewer studies have directly investigated whether AI chatbots may steer patients toward one treatment modality over another. This study therefore shifts the focus from ‘Is the answer generally correct?’ to ‘Does the AI-generated answer provide a clinically appropriate and ethically cautious treatment-direction recommendation?’ This distinction is important because treatment-direction advice may shape patient expectations before professional consultation.

The category-based findings should be interpreted cautiously because each category included only five scenarios, but they provide useful signals for risk-based AI communication. All models performed well in mild orthodontic problems, suggesting that AI chatbots may be more reliable when the scenario is simple and the treatment decision is relatively straightforward. In contrast, differences became more apparent in moderate, vertical, sagittal, and complex-risk scenarios. This pattern is consistent with the clinical nature of orthodontics: simple alignment questions are easier to answer generically, whereas complex malocclusions require individualized diagnosis and biomechanical planning. Therefore, AI chatbots may be most appropriate as supportive tools for basic patient education, not as independent decision aids for treatment selection.

### 4.3. Methodological Strengths, Limitations, and Future Directions

The moderate inter-expert agreement is a central limitation of the reference standard. Only 17 scenarios were unanimous, and one expert pair showed particularly low agreement. This likely reflects both genuine clinical preference variation and the information constraints of text-only vignettes. Importantly, the alternative dissenting-code analysis changed the descriptive ordering by placing Claude and Copilot at the same concordance rate and removed the significant omnibus difference. The majority code should therefore be understood as one transparent operational benchmark, not the unique correct treatment. The unanimous, individual-expert-support, and alternative-reference analyses provide complementary views of this uncertainty.

This study has several strengths. It used standardized clinical scenarios covering a broad range of orthodontic treatment-selection situations; compared four AI chatbots using the same patient-facing prompts under controlled querying conditions; used expert consensus from three orthodontic specialists as the reference benchmark; and coded AI-generated responses with two independent non-author orthodontists using a predefined framework. The study also evaluated communication-relevant safety outcomes, including potentially inappropriate treatment-direction advice, clear-aligner over-direction, overconfident wording, and referral to clinical examination.

Several limitations should be acknowledged. The fictional text-only vignettes cannot reproduce diagnosis from examination, radiographs, photographs, digital models, periodontal assessment, growth evaluation, or full patient preferences. Scenario development was investigator-led and did not include a formal content-validity index or independent lay-user pilot. The expert reference was moderately reliable rather than a gold standard, and results changed under the alternative dissenting-code definition. Each model-scenario pair was queried once with one Turkish wording on a single date; consequently, within-model stochasticity, prompt sensitivity, temporal drift, and cross-language variation were not estimated, and the reported confidence intervals do not include generation variance. Public interfaces and undisclosed backend assignments, particularly Copilot Smart mode, may change over time. Complete blinding to model identity was not guaranteed. The category analyses were underpowered and exploratory. Future work should use independently validated vignettes or complete de-identified clinical records with appropriate ethics oversight, repeated and paraphrased prompts, multilingual and longitudinal sampling, and direct assessment of patient preferences and decision behavior.

## 5. Conclusions

Under the sampled majority-benchmark conditions, the four user-facing chatbot interfaces produced different patterns of exact concordance and safety coding. However, moderate expert agreement and the non-significant alternative-reference analysis show that these estimates are conditional on how the expert benchmark is defined. The results should be interpreted as a snapshot of archived first responses, not as a stable ranking of underlying model capability.

AI-generated suggestions about clear aligners or fixed appliances require caution, particularly when a response gives definitive direction without examination, diagnostic records, or individualized planning. Patient-facing chatbots may support balanced pre-consultation education and help users prepare for professional consultation, but they should not replace diagnosis, risk assessment, or clinician-led shared decision-making. Responsible communication should preserve uncertainty, avoid premature steering, and prioritize appropriate referral.

## Figures and Tables

**Table 1:** Expert consensus distribution and inter-expert agreement.

Measure	Result
Consensus category 1: clear aligner more appropriate	7/40 (17.5%)
Consensus category 2: metal bracket/fixed appliance more appropriate	8/40 (20.0%)
Consensus category 3: both options may be appropriate	6/40 (15.0%)
Consensus category 4: examination required before decision	17/40 (42.5%)
Consensus category 5: advanced specialist/surgical evaluation required	2/40 (5.0%)
Complete agreement among three experts	17/40 (42.5%)
2/3 majority agreement	23/40 (57.5%)
Fleiss’ kappa	0.491
Gwet AC1	0.528
Mean observed agreement	0.617
Expert 1 vs. Expert 2 Cohen kappa	0.489
Expert 1 vs. Expert 3 Cohen kappa	0.757
Expert 2 vs. Expert 3 Cohen kappa	0.283

**Table 2:** Model-specific concordance and coded safety outcomes.

Model	Concordance n/N (%)	95% CI	Kappa (Bootstrap 95% CI)	Coded Safety Concern n/N (%)	95% CI	Referral to Examination n/N (%)	Overconfident Wording n/N (%)
ChatGPT	33/40 (82.5%)	68.1–91.3%	0.757 (0.586–0.897)	6/40 (15.0%)	7.1–29.1%	25/40 (62.5%)	4/40 (10.0%)
Claude	27/40 (67.5%)	52.0–79.9%	0.562 (0.361–0.748)	8/40 (20.0%)	10.5–34.8%	19/40 (47.5%)	4/40 (10.0%)
Copilot	17/40 (42.5%)	28.5–57.8%	0.294 (0.148–0.444)	17/40 (42.5%)	28.5–57.8%	3/40 (7.5%)	0/40 (0.0%)
Gemini	15/40 (37.5%)	24.2–53.0%	0.225 (0.087–0.388)	25/40 (62.5%)	47.0–75.8%	11/40 (27.5%)	25/40 (62.5%)

**Table 3:** Pairwise Holm-adjusted McNemar comparisons with paired risk differences and matched-pairs odds ratios.

Comparison	Exact Concordance Discordant Pairs; RD; mOR (95% CI); Adjusted *p*	Coded Safety Concern Discordant Pairs; RD; mOR (95% CI); Adjusted *p*
ChatGPT vs. Claude	Pairs 7;1	Pairs 3;5
	RD +15.0 pp	RD −5.0 pp
	mOR 7.00 (0.90–315.48)	mOR 0.60 (0.09–3.08)
	*p* 0.141	*p* 0.727
ChatGPT vs. Copilot	Pairs 17;1	Pairs 1;12
	RD +40.0 pp	RD −27.5 pp
	mOR 17.00 (2.66–710.46)	mOR 0.08 (0.002–0.56)
	*p* < 0.001	*p* 0.014
ChatGPT vs. Gemini	Pairs 18;0	Pairs 1;20
	RD +45.0 pp	RD −47.5 pp
	mOR ∞ (4.40–∞)	mOR 0.05 (0.001–0.31)
	*p* < 0.001	*p* < 0.001
Claude vs. Copilot	Pairs 12;2	Pairs 1;10
	RD +25.0 pp	RD −22.5 pp
	mOR 6.00 (1.34–55.20)	mOR 0.10 (0.002–0.70)
	*p* 0.039	*p* 0.035
Claude vs. Gemini	Pairs 12;0	Pairs 2;19
	RD +30.0 pp	RD −42.5 pp
	mOR ∞ (2.78–∞)	mOR 0.11 (0.01–0.44)
	*p* 0.002	*p* 0.001
Copilot vs. Gemini	Pairs 4;2	Pairs 2;10
	RD +5.0 pp	RD −20.0 pp
	mOR 2.00 (0.29–22.11)	mOR 0.20 (0.02–0.94)
	*p* 0.688	*p* 0.077

Note. Pairs are shown as first-model positive/second-model negative; second-model positive/first-model negative. RD = paired risk difference in percentage points (first minus second). mOR = matched-pairs odds ratio calculated from discordant cells, with exact 95% confidence interval. For concordance, mOR > 1 favors the first model; for safety concern, mOR < 1 indicates fewer concerns for the first model. Holm-adjusted *p* values are shown. Infinity is reported when the denominator discordant cell was zero.

**Table 4:** Sensitivity analysis according to expert agreement level.

Model	Unanimous Exact Concordance	Unanimous Coded Safety Concern	2/3 Majority Exact Concordance	2/3 Majority Coded Safety Concern
ChatGPT	17/17 (100.0%)	0/17 (0.0%)	16/23 (69.6%)	6/23 (26.1%)
Claude	14/17 (82.4%)	3/17 (17.6%)	13/23 (56.5%)	5/23 (21.7%)
Copilot	12/17 (70.6%)	5/17 (29.4%)	5/23 (21.7%)	12/23 (52.2%)
Gemini	11/17 (64.7%)	7/17 (41.2%)	4/23 (17.4%)	18/23 (78.3%)

**Table 5:** Distribution of expert consensus and AI treatment-direction recommendations.

Source/Model	Clear Aligner n (%)	Metal Bracket/Fixed Appliance n (%)	Both Options n (%)	Examination Required n (%)	Advanced/Surgical Evaluation n (%)
Expert consensus	7 (17.5%)	8 (20.0%)	6 (15.0%)	17 (42.5%)	2 (5.0%)
ChatGPT	11 (27.5%)	8 (20.0%)	2 (5.0%)	17 (42.5%)	2 (5.0%)
Claude	14 (35.0%)	6 (15.0%)	4 (10.0%)	14 (35.0%)	2 (5.0%)
Copilot	19 (47.5%)	15 (37.5%)	4 (10.0%)	1 (2.5%)	1 (2.5%)
Gemini	29 (72.5%)	6 (15.0%)	1 (2.5%)	3 (7.5%)	1 (2.5%)

Note. No AI-generated response was coded as unclear or not classifiable; therefore, code 6 is not shown as a separate column.

**Table 6:** Interpretive analysis separating exact concordance, non-safety-concern discordance, and potentially unsafe discordance.

Model	Exact Concordance n/N (%)	Discordant Without Coded Safety Concern n/N (%)	Potentially Unsafe Discordance n/N (%)	Responses Without Coded Safety Concern n/N (%)	Clear-Aligner Over-Direction n/N (%)
ChatGPT	33/40 (82.5%)	5/40 (12.5%)	2/40 (5.0%)	34/40 (85.0%)	4/40 (10.0%)
Claude	27/40 (67.5%)	6/40 (15.0%)	7/40 (17.5%)	32/40 (80.0%)	7/40 (17.5%)
Copilot	17/40 (42.5%)	6/40 (15.0%)	17/40 (42.5%)	23/40 (57.5%)	12/40 (30.0%)
Gemini	15/40 (37.5%)	2/40 (5.0%)	23/40 (57.5%)	15/40 (37.5%)	22/40 (55.0%)

Note. Coded safety concern and potentially unsafe discordance are related but not identical outcomes. A response could be exactly concordant with the expert consensus treatment-direction category but still receive a safety-concern code if the wording was overly definitive, insufficiently cautious, or clinically misleading.

**Table 7:** Descriptive subtypes of adjudicated coded safety concerns by model.

Safety-Concern Subtype or Context	ChatGPT	Claude	Copilot	Gemini
Total coded safety concerns	6	8	17	25
Clear-aligner over-direction among safety concerns	1	2	7	21
Under-referral for examination or advanced evaluation	2	5	16	15
Missed advanced/surgical referral	0	0	1	1
Overconfident wording flagged by either coder	2	3	0	19
Biomechanical/skeletal-category safety concerns (scenarios 11–25)	3	2	8	13
Risky/complex-category safety concerns (scenarios 31–35)	0	2	4	4

Note. The subtypes and category-context indicators were derived post hoc after adjudication from the final recommendation and safety codes, expert categories, scenario domains, and existing coder flags; they were not prespecified before data collection and should not be interpreted as confirmatory coding outcomes. Indicators were non-mutually exclusive; therefore, column counts do not sum to total safety concerns. Representative examples and operational coding definitions are provided in Supplementary Files S5 and S6.

**Table 8:** Exploratory category-based exact concordance with expert consensus.

Clinical Category	ChatGPT	Claude	Copilot	Gemini
Mild orthodontic problems	5/5	5/5	5/5	5/5
Moderate/borderline cases	5/5	3/5	1/5	1/5
Severe crowding and rotations	4/5	2/5	3/5	1/5
Vertical problems	3/5	3/5	1/5	0/5
Sagittal problems	4/5	4/5	1/5	1/5
Patient compliance and lifestyle factors	3/5	4/5	3/5	3/5
Risky/complex clinical conditions	5/5	3/5	1/5	2/5
Aesthetic expectations and patient preference	4/5	3/5	2/5	2/5

## Data Availability

The data supporting the findings of this study are contained within the article and Supplementary Files S1–S6.

## Supplementary Materials

The following supporting information can be downloaded at https://www.mdpi.com/article/10.3390/healthcare14162565/s1, Supplementary File S1: Standardized Clinical Scenarios and Patient-Facing Prompts; Supplementary File S2: Expert Consensus Reference Codes; Supplementary File S3: Original Turkish AI-Generated Responses; Supplementary File S4: Coded Dataset and Statistical Outputs; Supplementary File S5: AI Response Coding Framework; Supplementary File S6: Representative Translated Safety-Coding Examples.
