# Supplementary material for: Patient-Facing AI Chatbot Treatment-Direction Advice in Orthodontic Health Communication: A Scenario-Based Comparison with Expert Consensus
Source: Healthcare (Basel). 2026 Aug 16;14(16):2565. doi: 10.3390/healthcare14162565 (PMC13512535; doi:10.3390/healthcare14162565)
Supplement: Supplementary file 1 [file healthcare-14-02565-s001.zip › Supplementary File S1.pdf]

Supplementary File S1. Standardized Clinical Scenarios and Patient-Facing Prompts

This file provides the standardized clinical scenarios used for expert consensus coding and the corresponding patient-facing prompts used for chatbot querying. The English text is a translation prepared for international review. The original patient-facing prompts were submitted to the AI chatbots in Turkish.

The English text is provided only as a translation for international review. The original Turkish patient-facing prompts were the exact prompts submitted to the AI chatbots.

Scenario ID	Clinical category	Standardized clinical scenario (English translation)	Patient-facing prompt (English translation)	Original Turkish patient-facing prompt
1	Mild orthodontic problems	A 24-year-old adult patient in the permanent dentition with an Angle Class I molar relationship. Mild crowding of approximately 2-3 mm is present across the upper and lower arches. Overjet and overbite are within normal limits. The patient prefers an inconspicuous treatment option because of aesthetic concerns and states that they can wear aligners regularly.	I am 24 years old. I have very mild crowding, and I do not think I have a serious bite problem. I want a treatment that is not visible, and I think I can wear clear aligners regularly. In this situation, would clear aligners or metal brackets be more suitable for me?	24 yaşındayım. Dişlerimde çok hafif çapraşıklık var, kapanışımda ciddi bir sorun olduğunu düşünmüyorum. Görünmeyen bir tedavi istiyorum ve şeffaf plakları düzenli kullanabileceğimi düşünüyorum. Bu durumda benim için şeffaf plak mı yoksa metal braket mi daha uygun olur?
2	Mild orthodontic problems	A 29-year-old patient with mild anterior diastemas, no skeletal problem, and normal overjet and overbite. Oral hygiene is good, and the patient reports no periodontal problem. The patient is seeking an aesthetic treatment option because of professional/work-related concerns.	I am 29 years old. I have small spaces between my front teeth. I do not have gum problems and I pay attention to oral hygiene. Because of my work life, I want a more aesthetic treatment. Would clear aligners or metal brackets be more suitable?	29 yaşındayım. Ön dişlerimin arasında hafif boşluklar var. Diş eti problemim yok ve ağız hijyenime dikkat ediyorum. İş hayatım nedeniyle daha estetik bir tedavi istiyorum. Şeffaf plak mı yoksa metal braket mi daha uygun olur?
3	Mild orthodontic problems	A 21-year-old patient who previously underwent orthodontic treatment but developed mild relapse crowding in the lower anterior teeth after not wearing the retainer. Total crowding is approximately 2 mm. The patient wants a short and aesthetic correction.	I previously had braces, but because I did not wear my retainer regularly, my lower front teeth have shifted slightly. I only want a small correction. Would clear aligners be sufficient, or would metal brackets be needed?	Daha önce tel tedavisi görmüştüm ama pekiştirme plağımı düzenli kullanmadığım için alt ön dişlerimde hafif bozulma oldu. Sadece küçük bir düzeltme istiyorum. Bu durumda şeffaf plak yeterli olur mu, yoksa metal braket mi gerekir?
4	Mild orthodontic problems	A 26-year-old patient with mild rotation of the upper incisors and minimal lower anterior crowding. Occlusion appears generally stable. The patient reports good oral hygiene and states that they can comply with aligner wear.	I have mild rotations and a small amount of crowding in the lower front region. Overall, my bite seems good. I think I can wear aligners regularly. Would clear aligners or metal brackets be more suitable for me?	Dişlerimde çok ileri olmayan hafif dönüklükler ve alt ön bölgede az miktarda çapraşıklık var. Genel olarak dişlerimin kapanışı iyi görünüyor. Plakları düzenli kullanabileceğimi düşünüyorum. Benim için şeffaf plak mı metal braket mi daha uygun olur?
5	Mild orthodontic problems	An 18-year-old patient with mild crowding and mild aesthetic irregularity. There is no space deficiency suggesting a need for extraction. The patient is considering both clear aligners and metal brackets.	I am 18 years old. I have mild crowding, but I do not think it is very serious. I am interested in both clear aligners and metal brackets. In such a case, which option might be more suitable?	18 yaşındayım. Dişlerimde hafif çapraşıklık var ama çok ciddi bir sorun olduğunu düşünmüyorum. Hem şeffaf plak hem de metal braket seçeneğini merak ediyorum. Böyle bir durumda hangisi daha uygun olabilir?

Scenario ID	Clinical category	Standardized clinical scenario (English translation)	Patient-facing prompt (English translation)	Original Turkish patient-facing prompt
6	Moderate/borderline cases	A 17-year-old patient with 4 mm crowding in the upper arch and 5 mm crowding in the lower arch. An Angle Class I relationship is present. Overjet and overbite are close to normal limits. The patient wants aesthetic treatment but does not want the treatment duration to be prolonged.	I am 17 years old. I have moderate crowding in my upper and lower teeth. I do not know whether tooth extraction is needed. I want clear aligners for aesthetic reasons, but I also do not want the treatment to take too long. Which method would be more suitable for me?	17 yaşındayım. Üst ve alt dişlerimde orta düzeyde çapraşıklık var. Diş çekimi gerekip gerekmediğini bilmiyorum. Estetik olarak şeffaf plak istiyorum ama tedavinin uzamasını da istemiyorum. Benim için hangi yöntem daha uygun olur?
7	Moderate/borderline cases	A 25-year-old patient with moderate anterior crowding and mild arch constriction. Whether extraction is required cannot be determined without clinical examination and model analysis. The patient asks whether the condition can be corrected with clear aligners.	I am 25 years old. I think I have moderate crowding and some narrowing. I am not sure whether this can be corrected with clear aligners. In this situation, would clear aligners or metal brackets be more appropriate?	25 yaşındayım. Dişlerimde orta düzeyde çapraşıklık ve biraz darlık olduğunu düşünüyorum. Şeffaf plakla düzelip düzelmeyeceğinden emin değilim. Bu durumda şeffaf plak mı yoksa metal braket mi daha doğru olur?
8	Moderate/borderline cases	A 19-year-old patient with mild rotations of the upper anterior teeth and moderate crowding in the lower anterior region. The patient wants clear aligners for aesthetic reasons but says they are unsure whether they can wear them for 20-22 hours per day.	I am 19 years old. My front teeth are rotated and my lower teeth are crowded. I want clear aligners, but honestly I am not sure whether I can wear them for 20-22 hours a day. Would clear aligners be suitable, or would metal brackets make more sense?	19 yaşındayım. Ön dişlerimde dönüklükler ve alt dişlerimde çapraşıklık var. Şeffaf plak istiyorum ama açıkçası günde 20–22 saat takabilir miyim emin değilim. Bu durumda şeffaf plak uygun olur mu, yoksa metal braket daha mı mantıklı olur?
9	Moderate/borderline cases	A 32-year-old patient with mild-to-moderate crowding who reports a periodontal history and gingival recession on some teeth. The patient asks whether clear aligners or brackets would be safer.	I am 32 years old. I have mild-to-moderate crowding, but I also have gum recession on some teeth. Would clear aligners or bracket treatment be safer for me?	32 yaşındayım. Dişlerimde hafif-orta çapraşıklık var ama bazı dişlerimde diş eti çekilmesi de bulunuyor. Şeffaf plak mı yoksa braket tedavisi mi benim için daha güvenli olur?
10	Moderate/borderline cases	A 16-year-old patient with moderate crowding, mildly increased overjet, and good oral hygiene. The patient participates in sports and is concerned that brackets may cause trauma during sports activities.	I am 16 years old. I have moderate crowding and I play sports. I am worried that metal brackets may injure my lip or cheek during sports. In this situation, would clear aligners or metal brackets be more suitable?	16 yaşındayım. Dişlerimde orta düzeyde çapraşıklık var ve spor yapıyorum. Metal braketlerin spor sırasında dudağıma veya yanağıma zarar vermesinden endişeleniyorum. Bu durumda şeffaf plak mı metal braket mi daha uygun olur?
11	Severe crowding and rotations	A 15-year-old patient with 8 mm crowding in the upper arch and 7 mm crowding in the lower arch. Space deficiency for the canines is evident, and some teeth show severe rotations. The patient states that they want treatment only with clear aligners.	I am 15 years old. I have severe crowding; especially, it seems there is no space for my canine teeth, and some teeth are very rotated. I only want to use clear aligners. Would clear aligners be sufficient in this situation?	15 yaşındayım. Dişlerimde ciddi çapraşıklık var, özellikle köpek dişlerime yer yok gibi görünüyor ve bazı dişlerim çok dönük. Ben sadece şeffaf plak kullanmak istiyorum. Bu durumda şeffaf plak yeterli olur mu?
12	Severe crowding and rotations	A 22-year-old patient with marked rotations of the upper lateral incisors and severe lower incisor crowding. Extraction treatment is considered possible because of arch length deficiency. The patient asks whether clear aligners would be sufficient.	I am 22 years old. I have marked rotations in my front teeth and severe crowding in my lower teeth. I was told that tooth extraction may be needed, but I am not sure. In such a situation, would clear aligners or metal brackets be more suitable?	22 yaşındayım. Ön dişlerimde belirgin dönüklükler ve alt dişlerimde ileri çapraşıklık var. Diş çekimi gerekebilir denildi ama emin değilim. Böyle bir durumda şeffaf plak mı metal braket mi daha uygun olur?
13	Severe crowding and rotations	A 14-year-old patient in the permanent dentition with one	I am 14 years old. I was told that one of my canine teeth is not in its proper	14 yaşındayım. Bir köpek dişimin yerinde olmadığı ve yukarıda kaldığı

Scenario ID	Clinical category	Standardized clinical scenario (English translation)	Patient-facing prompt (English translation)	Original Turkish patient-facing prompt
		maxillary canine in an ectopic position and space deficiency in the arch. The patient asks whether the canine can be brought into position with clear aligners.	place and is positioned high. I wonder whether this tooth can be brought into place with clear aligners. Are clear aligners suitable, or would metal brackets be needed?	söylendi. Ben bu dişin şeffaf plakla yerine gelip gelemeyeceğini merak ediyorum. Şeffaf plak uygun olur mu, yoksa metal braket mi gerekir?
14	Severe crowding and rotations	A 28-year-old patient with severe crowding in the lower anterior region and marked rotations in multiple teeth. Oral hygiene is moderate. The patient wants the fastest possible treatment option.	I am 28 years old. My lower front teeth are very crowded and some teeth are quite rotated. I want the treatment to be as fast as possible. Would clear aligners or metal brackets make more sense?	28 yaşındayım. Alt ön dişlerim çok çapraşık ve bazı dişlerim oldukça dönük. Tedavinin mümkün olduğunca hızlı olmasını istiyorum. Şeffaf plak mı yoksa metal braket mi daha mantıklı olur?
15	Severe crowding and rotations	A 20-year-old patient with severe crowding in the upper and lower arches, a narrow arch form, and aesthetic expectations. It is unclear whether interproximal reduction, expansion, or extraction would be required.	I am 20 years old. I have marked crowding in my upper and lower teeth, my jaw seems a little narrow, and I am bothered aesthetically. I do not know whether extraction, interproximal reduction, or expansion would be needed. Would clear aligners or metal brackets be more suitable?	20 yaşındayım. Üst ve alt dişlerimde belirgin çapraşıklık var, çenem biraz dar görünüyor ve estetik olarak rahatsız oluyorum. Diş çekimi, dişler arası aşındırma veya genişletme gerekir mi bilmiyorum. Şeffaf plak mı metal braket mi daha uygun olur?
16	Vertical problems	An 18-year-old patient with a pronounced deep bite; the lower incisors are reported to contact the palatal mucosa. Lower incisor intrusion and bite opening may be required. The patient wants clear aligner treatment.	I am 18 years old. When I close my teeth, my upper teeth cover my lower teeth too much; it even feels as if my lower teeth touch my palate. I want clear aligners. In this situation, would clear aligners or metal brackets be more suitable?	18 yaşındayım. Dişlerimi kapattığımda üst dişlerim alt dişlerimi çok fazla örtüyor, hatta alt dişlerim damağıma değiyor gibi oluyor. Şeffaf plak istiyorum. Bu durumda şeffaf plak mı metal braket mi daha uygun olur?
17	Vertical problems	A 23-year-old patient with a mild deep bite and mild crowding. No skeletal problem has been reported. The patient seeks both an aesthetic and comfortable treatment option.	I am 23 years old. I have a mild deep bite and a little crowding. I do not think my problem is very complex. I want a treatment that is both aesthetic and comfortable. Would clear aligners or metal brackets be more suitable?	23 yaşındayım. Hafif derin kapanışım ve biraz çapraşıklığım var. Çok karmaşık bir problemim olduğunu düşünmüyorum. Hem estetik hem konforlu bir tedavi istiyorum. Şeffaf plak mı metal braket mi daha uygun olabilir?
18	Vertical problems	A 16-year-old patient with anterior open bite who reports a tongue-thrust habit. Posterior contacts are present, but anterior closure cannot be achieved.	I am 16 years old. My front teeth do not close, and I was told that I push my tongue between my teeth. Can I be treated with clear aligners, or would metal brackets be needed?	16 yaşındayım. Ön dişlerim kapanmıyor ve dilimi dişlerimin arasına ittiğim söyleniyor. Bu durumda şeffaf plakla tedavi olabilir miyim, yoksa metal braket mi gerekir?
19	Vertical problems	A 27-year-old patient with mild anterior open bite and good oral hygiene. The patient asks whether clear aligners can close the opening. It is unknown whether a tongue habit or skeletal problem is present.	I am 27 years old. I have a slight opening between my front teeth when I bite. I wonder whether clear aligners can close it. I do not know whether I have a tongue habit or jaw problem. Which treatment would be more suitable?	27 yaşındayım. Ön dişlerim arasında kapanışta hafif açıklık var. Şeffaf plakların bunu kapatıp kapatamayacağını merak ediyorum. Dil alışkanlığı veya çene problemim olup olmadığını bilmiyorum. Hangi tedavi daha uygun olur?
20	Vertical problems	A 14-year-old patient with severe deep bite. Increased lower facial height is reportedly absent, but detailed cephalometric evaluation has not been performed. The patient does not want metal brackets.	I am 14 years old. When I close my teeth, my lower teeth are almost not visible, so I have a deep bite. I do not want metal brackets. Can this be corrected with clear aligners?	14 yaşındayım. Dişlerimi kapattığımda alt dişlerim neredeyse görünmüyor, yani derin kapanışım var. Metal braket istemiyorum. Şeffaf plakla düzelebilir mi?
21	Sagittal problems	A 15-year-old patient with an Angle Class II molar relationship and	I am 15 years old. My upper teeth are positioned forward, and I was	15 yaşındayım. Üst dişlerim önde duruyor ve kapanışımda Sınıf II

Scenario ID	Clinical category	Standardized clinical scenario (English translation)	Patient-facing prompt (English translation)	Original Turkish patient-facing prompt
		increased overjet. The upper incisors appear protrusive. Growth status is unknown. The patient asks whether the problem can be corrected with clear aligners.	told that I may have a Class II bite. I do not know whether I am still growing. Can this be corrected with clear aligners, or would metal brackets be needed?	denilen bir durum olabileceği söylendi. Büyümem devam ediyor mu bilmiyorum. Şeffaf plakla düzelir mi, yoksa metal braket mi gerekir?
22	Sagittal problems	A 19-year-old patient with a mild Class II relationship, minimal crowding, and good oral hygiene. The patient wants aesthetic treatment and states that they can comply with elastic wear if needed.	I am 19 years old. I have a mild Class II bite, but my teeth are not very crowded. I think I can wear elastics if necessary. I want an aesthetic treatment. Would clear aligners or metal brackets be more suitable?	19 yaşındayım. Hafif Sınıf II kapanışım var ama dişlerimde çok fazla çapraşıklık yok. Gerekirse lastik kullanabileceğimi düşünüyorum. Estetik bir tedavi istiyorum. Şeffaf plak mı metal braket mi daha uygun olur?
23	Sagittal problems	A 17-year-old patient with a Class II relationship in which maxillary molar distalization may be needed. Anchorage requirements and third molar status are unknown. The patient asks whether clear aligners or brackets would be more appropriate.	I am 17 years old. I was told that my upper molars may need to be moved backward. I do not know whether clear aligners or metal brackets are more suitable for this. Which method might be more appropriate?	17 yaşındayım. Üst azı dişlerimin geriye alınması gerekebilir denildi. Bunun için şeffaf plak mı metal braket mi daha uygun olur bilmiyorum. Hangi yöntem daha doğru olabilir?
24	Sagittal problems	A 21-year-old patient with a mild Class III tendency and anterior edge-to-edge bite. The mandible appears prominent, but cephalometric analysis has not been performed. The patient wants only aesthetic clear aligner treatment.	I am 21 years old. My lower jaw seems a little forward and my front teeth meet edge-to-edge. Cephalometric analysis has not been performed. I want to improve the appearance only with clear aligners. Is this possible?	21 yaşındayım. Alt çenem biraz önde gibi duruyor ve ön dişlerim baş başa kapanıyor. Sefalometrik analiz yapılmadı. Ben sadece şeffaf plakla estetik olarak düzelmek istiyorum. Bu mümkün olur mu?
25	Sagittal problems	An 18-year-old patient with an apparent skeletal Class III pattern, reverse overjet, and mandibular prominence in the facial profile. The patient asks whether correction is possible without surgery and preferably with clear aligners.	I am 18 years old. My lower jaw is clearly forward, my front teeth have a reverse bite, and I am unhappy with my profile. I want correction without surgery and, if possible, with clear aligners. Which treatment would be more suitable in this situation?	18 yaşındayım. Alt çenem belirgin şekilde önde, ön dişlerim ters kapanıyor ve profilimden rahatsızım. Ameliyatsız ve mümkünse şeffaf plakla düzelmek istiyorum. Bu durumda hangi tedavi daha uygun olur?
26	Patient compliance and lifestyle factors	A 20-year-old patient with mild-to-moderate crowding. The patient has high aesthetic expectations but says they may forget to wear aligners during the day and may not always be able to brush after meals.	I am 20 years old. I have mild-to-moderate crowding. I want clear aligners aesthetically, but I may forget to wear them during the day and I may not always brush after meals. Would clear aligners still be suitable?	20 yaşındayım. Dişlerimde hafif-orta çapraşıklık var. Estetik olarak şeffaf plak istiyorum ama gün içinde takmayı unutabilirim ve yemeklerden sonra her zaman diş fırçalayamayabilirim. Yine de şeffaf plak uygun olur mu?
27	Patient compliance and lifestyle factors	A 30-year-old patient with mild crowding and good oral hygiene. The patient states that they are highly disciplined, can wear aligners for 22 hours per day, and can attend appointments regularly.	I am 30 years old. I have mild crowding and I pay attention to oral hygiene. I think I can wear aligners for 22 hours a day and attend my appointments regularly. Would clear aligners or metal brackets be more suitable?	30 yaşındayım. Dişlerimde hafif çapraşıklık var ve ağız hijyenime dikkat ediyorum. Plakları günde 22 saat kullanabileceğimi ve kontrollerime düzenli gelebileceğimi düşünüyorum. Şeffaf plak mı metal braket mi daha uygun olur?
28	Patient compliance and lifestyle factors	A 16-year-old patient with moderate crowding. The family wants clear aligner treatment, but the patient says they do not want to wear aligners regularly at school.	I am 16 years old. I have moderate crowding. My family wants clear aligners, but I do not want to wear aligners at school and I am not sure whether I can use them regularly. Which treatment would be more suitable?	16 yaşındayım. Dişlerimde orta düzeyde çapraşıklık var. Ailem şeffaf plak istiyor ama ben okulda plak takmak istemiyorum ve düzenli kullanıp kullanamayacağımdan emin değilim. Hangi tedavi daha uygun olur?

Scenario ID	Clinical category	Standardized clinical scenario (English translation)	Patient-facing prompt (English translation)	Original Turkish patient-facing prompt
29	Patient compliance and lifestyle factors	A 35-year-old patient with mild dental irregularity. The patient travels frequently and says they may miss appointments. How treatment follow-up would be maintained is unclear.	I am 35 years old. I have mild irregularity in my teeth, but I travel frequently for work and may miss appointments. Would clear aligners or metal brackets be more suitable for me?	35 yaşındayım. Dişlerimde hafif düzensizlik var ama işim nedeniyle sık seyahat ediyorum ve randevuları aksatabilirim. Şeffaf plak mı metal braket mi benim için daha uygun olur?
30	Patient compliance and lifestyle factors	A 22-year-old patient with mild crowding and aesthetic concerns. The patient plays a musical instrument and is worried about the effect of the appliance during speech and performance.	I am 22 years old. I have mild crowding and aesthetic concerns. I also play a musical instrument, and I am worried whether treatment will affect my speech and performance. Which treatment would be more suitable?	22 yaşındayım. Dişlerimde hafif çapraşıklık var ve estetik kaygım var. Aynı zamanda müzik aleti çalıyorum, konuşma ve performans sırasında tedavinin beni etkileyip etkilemeyeceğinden endişeliyim. Hangi tedavi daha uygun olur?
31	Risky/complex clinical conditions	A 38-year-old patient who previously underwent periodontal treatment and reports mobility and gingival recession in some teeth. The patient has crowding and wants it corrected quickly with clear aligners.	I am 38 years old. I previously had gum treatment; some of my teeth are mobile and have gum recession. I have crowding and I want it corrected quickly with clear aligners. Would this be suitable for me?	38 yaşındayım. Daha önce diş eti tedavisi gördüm, bazı dişlerimde sallanma ve diş eti çekilmesi var. Dişlerimde çapraşıklık olduğu için şeffaf plakla hızlıca düzelmesini istiyorum. Bu benim için uygun olur mu?
32	Risky/complex clinical conditions	A 24-year-old patient who reports that root resorption occurred during previous orthodontic treatment. The patient now has relapse and asks which method would be safer for retreatment.	I am 24 years old. I was told that I had root resorption during previous orthodontic treatment. Now my teeth have shifted again. Would clear aligners or metal brackets be safer?	24 yaşındayım. Daha önce ortodontik tedavi sırasında kök erimesi yaşadığımı söylediler. Şimdi dişlerimde tekrar bozulma oldu. Şeffaf plak mı metal braket mi daha güvenli olur?
33	Risky/complex clinical conditions	A 29-year-old patient who reports temporomandibular joint pain, clicking, and occasional limitation in mouth opening. The patient also has crowding and asks whether clear aligners can solve the joint problem.	I am 29 years old. I have pain and clicking in my jaw joint, and sometimes I have difficulty opening my mouth. I also have crowding. Can clear aligners solve my joint problem, and which treatment would be more suitable?	29 yaşındayım. Çene eklemimde ağrı ve klik sesi var, bazen ağzımı açmakta zorlanıyorum. Dişlerimde de çapraşıklık var. Şeffaf plaklar eklem sorunumu çözer mi ve hangi tedavi daha uygun olur?
34	Risky/complex clinical conditions	An 18-year-old patient with a suspected impacted canine. A dentist requested a panoramic radiograph, but imaging has not yet been performed. The patient wants clear aligner treatment for aesthetic reasons.	I am 18 years old. I was told that one of my canine teeth may be impacted, and a panoramic radiograph was requested, but I have not had it taken yet. I want clear aligners because they are aesthetic. Are clear aligners suitable in this situation?	18 yaşındayım. Bir köpek dişimin gömülü olabileceği söylendi ve panoramik film istenmişti ama henüz çektirmedim. Estetik olduğu için şeffaf plak istiyorum. Bu durumda şeffaf plak uygun olur mu?
35	Risky/complex clinical conditions	A 26-year-old patient with anterior crowding, a missing tooth, and a future implant plan. The patient asks whether orthodontic treatment can create space before implant placement.	I am 26 years old. I have crowding in my front teeth, I also have a missing tooth, and an implant is planned in the future. Can orthodontic treatment create space for the implant? Would clear aligners or metal brackets be more suitable?	26 yaşındayım. Ön dişlerimde çapraşıklık var, ayrıca eksik dişim var ve ileride implant planlanıyor. Ortodontiyle implant için yer açılabilir mi? Şeffaf plak mı metal braket mi daha uygun olur?
36	Aesthetic expectations and patient preference	A 23-year-old patient with mild crowding who specifically requests an inconspicuous treatment option. Clinical findings appear simple, oral hygiene is good, and the patient is motivated to wear aligners regularly.	I am 23 years old. I have mild crowding and specifically want an invisible treatment. My oral hygiene is good and I think I can wear aligners regularly. Are clear aligners suitable for me?	23 yaşındayım. Dişlerimde hafif çapraşıklık var ve özellikle görünmeyen bir tedavi istiyorum. Ağız hijyenim iyi ve plakları düzenli kullanabileceğimi düşünüyorum. Benim için şeffaf plak uygun olur mu?

Scenario ID	Clinical category	Standardized clinical scenario (English translation)	Patient-facing prompt (English translation)	Original Turkish patient-facing prompt
37	Aesthetic expectations and patient preference	A 17-year-old patient with severe crowding and marked rotations. For social reasons, the patient strongly refuses metal brackets and says they will accept only clear aligners.	I am 17 years old. I have severe crowding and marked rotations, but for social reasons I definitely do not want metal brackets. I only want to use clear aligners. Can I be treated with clear aligners in this situation?	17 yaşındayım. Dişlerimde ciddi çapraşıklık ve belirgin dönüklükler var ama sosyal nedenlerle kesinlikle metal braket istemiyorum. Sadece şeffaf plak kullanmak istiyorum. Bu durumda şeffaf plakla tedavi olabilir miyim?
38	Aesthetic expectations and patient preference	A 28-year-old patient with moderate crowding, mild deep bite, and high aesthetic expectations. The patient wants clear aligners because of professional/work-related reasons but accepts that clinical suitability should be determined by examination.	I am 28 years old. I have moderate crowding and a mild deep bite. I want clear aligners because of my work life, but I know that suitability should be determined by examination. Which treatment might be more suitable?	28 yaşındayım. Dişlerimde orta düzey çapraşıklık ve hafif derin kapanış var. İş hayatım nedeniyle şeffaf plak istiyorum ama uygun olup olmadığının muayeneyle anlaşılacağını biliyorum. Sizce hangi tedavi daha uygun olabilir?
39	Aesthetic expectations and patient preference	A 21-year-old patient with mild spacing between the anterior teeth and aesthetic concerns. The patient wants treatment to be as inconspicuous as possible. Jaw relationship and occlusion appear normal.	I am 21 years old. I have small spaces between my front teeth and I am bothered aesthetically. I want the treatment to be as inconspicuous as possible. I do not think I have a serious bite problem. Would clear aligners or metal brackets be more suitable?	21 yaşındayım. Ön dişlerimin arasında hafif boşluklar var ve estetik olarak rahatsız oluyorum. Tedavinin mümkün olduğunca az belli olmasını istiyorum. Kapanışımda ciddi bir sorun olduğunu düşünmüyorum. Şeffaf plak mı metal braket mi daha uygun olur?
40	Aesthetic expectations and patient preference	A 19-year-old patient with marked skeletal asymmetry, chin deviation, and suspected crossbite. The patient does not want surgery and wants aesthetic improvement only with clear aligners.	I am 19 years old. I have jaw asymmetry, my chin seems to deviate to one side, and I was told I may have a crossbite. I do not want surgery; I only want aesthetic improvement with clear aligners. Which treatment would be more suitable in this situation?	19 yaşındayım. Çenemde asimetri var, çene ucum bir tarafa kayıyor gibi ve çapraz kapanış olabileceği söylendi. Cerrahi istemiyorum, sadece şeffaf plakla estetik düzelme istiyorum. Bu durumda hangi tedavi daha uygun olur?
