# Supplementary material for: Patient-Facing AI Chatbot Treatment-Direction Advice in Orthodontic Health Communication: A Scenario-Based Comparison with Expert Consensus
Source: Healthcare (Basel). 2026 Aug 16;14(16):2565. doi: 10.3390/healthcare14162565 (PMC13512535; doi:10.3390/healthcare14162565)
Supplement: Supplementary file 1 [file healthcare-14-02565-s001.zip › Supplementary File S2.pdf]

Supplementary File S2. Expert Consensus Reference Codes

Three orthodontic specialists independently coded each standardized scenario. The final reference category was determined by majority agreement. This file reports individual expert codes, final consensus codes, English labels, and agreement type.

Treatment-direction code legend

Code	Meaning
1	Clear aligner therapy may be more appropriate
2	Metal bracket/fixed appliance treatment may be more appropriate
3	Both treatment options may be appropriate
4	No decision can be made without clinical examination
5	Advanced specialist and/or surgical evaluation is required

Inter-expert agreement summary

Measure	Result
Complete agreement among three experts	17/40 (42.5%)
2/3 majority agreement	23/40 (57.5%)
No majority / consensus discussion required	0/40 (0.0%)
Fleiss kappa	0.491
Gwet AC1	0.528
Mean observed agreement	0.617
Expert 1 vs Expert 2 Cohen kappa	0.489
Expert 1 vs Expert 3 Cohen kappa	0.757
Expert 2 vs Expert 3 Cohen kappa	0.283

Scenario-level expert consensus codes

Scenario ID	Clinical category	Expert 1 code	Expert 2 code	Expert 3 code	Final consensus code	Final consensus label	Agreement type
1	Mild orthodontic problems	1	1	1	1	Clear aligner therapy may be more appropriate	Unanimous
2	Mild orthodontic problems	1	1	1	1	Clear aligner therapy may be more appropriate	Unanimous
3	Mild orthodontic problems	1	1	1	1	Clear aligner therapy may be more appropriate	Unanimous
4	Mild orthodontic problems	1	1	1	1	Clear aligner therapy may be more appropriate	Unanimous
5	Mild orthodontic problems	3	1	3	3	Both treatment options may be appropriate	2/3 majority
6	Moderate/borderline cases	4	3	4	4	No decision can be made without clinical examination	2/3 majority
7	Moderate/borderline cases	4	4	3	4	No decision can be made without clinical examination	2/3 majority

Scenario ID	Clinical category	Expert 1 code	Expert 2 code	Expert 3 code	Final consensus code	Final consensus label	Agreement type
8	Moderate/borderline cases	2	2	3	2	Metal bracket/fixed appliance treatment may be more appropriate	2/3 majority
9	Moderate/borderline cases	4	4	4	4	No decision can be made without clinical examination	Unanimous
10	Moderate/borderline cases	3	1	3	3	Both treatment options may be appropriate	2/3 majority
11	Severe crowding and rotations	2	2	4	2	Metal bracket/fixed appliance treatment may be more appropriate	2/3 majority
12	Severe crowding and rotations	4	2	4	4	No decision can be made without clinical examination	2/3 majority
13	Severe crowding and rotations	2	2	2	2	Metal bracket/fixed appliance treatment may be more appropriate	Unanimous
14	Severe crowding and rotations	2	2	2	2	Metal bracket/fixed appliance treatment may be more appropriate	Unanimous
15	Severe crowding and rotations	4	2	4	4	No decision can be made without clinical examination	2/3 majority
16	Vertical problems	2	2	4	2	Metal bracket/fixed appliance treatment may be more appropriate	2/3 majority
17	Vertical problems	3	1	3	3	Both treatment options may be appropriate	2/3 majority
18	Vertical problems	4	4	5	4	No decision can be made without clinical examination	2/3 majority
19	Vertical problems	4	3	4	4	No decision can be made without clinical examination	2/3 majority
20	Vertical problems	4	2	4	4	No decision can be made without clinical examination	2/3 majority
21	Sagittal problems	4	3	4	4	No decision can be made without clinical examination	2/3 majority
22	Sagittal problems	3	1	3	3	Both treatment options may be appropriate	2/3 majority
23	Sagittal problems	4	2	4	4	No decision can be made without clinical examination	2/3 majority

Scenario ID	Clinical category	Expert 1 code	Expert 2 code	Expert 3 code	Final consensus code	Final consensus label	Agreement type
24	Sagittal problems	4	5	4	4	No decision can be made without clinical examination	2/3 majority
25	Sagittal problems	5	5	5	5	Advanced specialist and/or surgical evaluation is required	Unanimous
26	Patient compliance and lifestyle factors	2	2	4	2	Metal bracket/fixed appliance treatment may be more appropriate	2/3 majority
27	Patient compliance and lifestyle factors	1	1	1	1	Clear aligner therapy may be more appropriate	Unanimous
28	Patient compliance and lifestyle factors	2	2	2	2	Metal bracket/fixed appliance treatment may be more appropriate	Unanimous
29	Patient compliance and lifestyle factors	4	3	4	4	No decision can be made without clinical examination	2/3 majority
30	Patient compliance and lifestyle factors	3	1	3	3	Both treatment options may be appropriate	2/3 majority
31	Risky/complex clinical conditions	4	4	4	4	No decision can be made without clinical examination	Unanimous
32	Risky/complex clinical conditions	4	4	4	4	No decision can be made without clinical examination	Unanimous
33	Risky/complex clinical conditions	4	4	4	4	No decision can be made without clinical examination	Unanimous
34	Risky/complex clinical conditions	4	5	4	4	No decision can be made without clinical examination	2/3 majority
35	Risky/complex clinical conditions	4	4	4	4	No decision can be made without clinical examination	Unanimous
36	Aesthetic expectations and patient preference	1	1	1	1	Clear aligner therapy may be more appropriate	Unanimous
37	Aesthetic expectations and patient preference	2	2	4	2	Metal bracket/fixed appliance treatment may be more appropriate	2/3 majority
38	Aesthetic expectations and patient preference	3	4	3	3	Both treatment options may be appropriate	2/3 majority
39	Aesthetic expectations and patient preference	1	1	1	1	Clear aligner therapy may be more appropriate	Unanimous
40	Aesthetic expectations and patient preference	5	5	5	5	Advanced specialist and/or surgical evaluation is required	Unanimous
